# Non-contrast CT-based radiomics nomogram of pericoronary adipose tissue for predicting haemodynamically significant coronary stenosis in patients with type 2 diabetes

**DOI:** 10.1186/s12880-023-01051-0

**Published:** 2023-07-28

**Authors:** Can Chen, Meng Chen, Qing Tao, Su Hu, Chunhong Hu

**Affiliations:** Department of Radiology, The First Affiliated Hospital of Soochow University, Suzhou, 215006 Jiangsu Province China

**Keywords:** Non-contrast CT, Pericoronary adipose tissue, Radiomics, Nomogram, CCTA derived fractional flow reserve, Haemodynamically significant coronary stenosis

## Abstract

**Background:**

Type 2 diabetes mellitus (T2DM) patients have a higher incidence of coronary artery disease than the general population. The aim of this study was to develop a radiomics nomogram of pericoronary adipose tissue (PCAT) based on non-contrast CT to predict haemodynamically significant coronary stenosis in T2DM patients.

**Methods:**

The study enrolled 215 T2DM patients who underwent non-contrast CT and coronary computed tomography angiography (CCTA). CCTA derived fractional flow reserve (FFR_CT_) ≤ 0.80 was defined as hemodynamically significant stenosis.1691 radiomics features were extracted from PCAT on non-contrast CT. Minimum redundancy maximum relevance (mRMR) and least absolute shrinkage and selection operator (LASSO) were used to select useful radiomics features to construct Radscore. Logistic regression was applied to select significant factors among Radscore, fat attenuation index (FAI) and coronary artery calcium score (CACS) to construct radiomics nomogram.

**Results:**

Radscore [odds ratio (OR) = 2.84; *P* < 0.001] and CACS (OR = 1.00; *P* = 0.023) were identified as independent predictors to construct the radiomics nomogram. The radiomics nomogram showed excellent performance [training cohort: area under the curve (AUC) = 0.81; 95% CI: 0.76–0.86; validation cohort: AUC = 0.83; 95%CI: 0.76–0.90] to predict haemodynamically significant coronary stenosis in patients with T2DM. Decision curve analysis demonstrated high clinical value of the radiomics nomogram.

**Conclusion:**

The non-contrast CT-based radiomics nomogram of PCAT could effectively predict haemodynamically significant coronary stenosis in patients with T2DM, which might be a potential noninvasive tool for screening of high-risk patients.

## Introduction

Coronary artery disease (CAD) is the most important cardiovascular disease threatening human health [1]. Type 2 diabetes mellitus (T2DM) is significantly associated with increased risk of CAD, and morbidity and mortality of patients with CAD is considerably higher in the presence of diabetes [2–6]. The treatment strategies for obstructive CAD vary depending on whether the lesion is hemodynamically significant. Therefore, early screening and prediction of high-risk CAD with haemodynamically significant stenosis in T2DM patients can reduce the occurrence of major adverse cardiovascular events (MACE), which has important clinical value. Fractional flow reserve (FFR) is the gold standard for diagnosing hemodynamically significant coronary stenosis, while it is difficult to access due to high costs and potential risks, limiting its widespread use [7–10]. Coronary CT angiography (CCTA) derived fractional flow reserve (FFR_CT_), has highly consistent assessment in myocardial ischemia compared with invasive FFR [11–13], without extra image acquisition and taking adenosine, showing great potential in the diagnosis of functional myocardial ischemia.

Inflammatory response of the coronary arteries has been shown to affect the formation and differentiation of pericoronary adipose tissue (PCAT) by releasing cytokines to prevent the lipid accumulation, which can be indicated by fat attenuation index (FAI) [14]. Meanwhile, chronic atherosclerosis and vascular inflammation of coronary artery can trigger permanent changes in the perivascular space, including fibrosis and microvascular remodeling [15, 16], which can be captured by radiomics with high-throughput extraction of quantitative features [17].Recently, radiomic signatures of PCAT have been proved to have important value for predicting hemodynamic significance of coronary stenosis [18–20], however above studies mainly focused on radiomics analysis based CCTA. The use of iodine contrast agents intravenously might lead to the risk of allergic reaction, renal impairment and microcirculation disorders. Hence, this study aimed to develop a radiomics nomogram of PCAT based on non-contrast CT to predict haemodynamically significant coronary stenosis in patients with T2DM.

## Material and methods

Local institutional review board and the ethics committee approved this study, and the requirement to acquire informed consent was waived.

### Patients

T2DM patients with suspected CAD who underwent non-contrast CT scan [coronary artery calcium score (CACS) scan] and CCTA from January 2020 to September 2022 were initially included in this study. The exclusion criteria were as follows: (1) patients without T2DM; (2) patients with previous history of CAD; (3) patients with history of cardiac or coronary surgery, including permanent pacemaker placement, cardiac valve replacement, percutaneous coronary intervention (PCI) and coronary artery bypass grafting (CABG), etc.; (4) patients with serious life-threatening diseases; (5) anomalous origin of coronary artery, coronary malformation or aneurysm; (6) poor image quality. Patients were excluded if any of above criteria was met. Clinical characteristics were collected from the medical records. Finally, 215 patients with 514 vessels were included, and vessels were divided into the training and validation cohorts at a ratio of 7:3.

### CT images acquisition protocol

All image acquisitions were performed using a 256 row CT scanner (Revolution CT, GE Healthcare, Milwaukee). Patients received oral beat-blockers when heart rate was over 70 beat/min. Before CT scan, each patient received sublingual nitroglycerin for vasodilation. non-contrast CT scan and CCTA scan were performed for each patient. Scan parameters were shown in Table 1. Forty-five 45 milliliter of iodized contrast agent iodixanol (370 mg/ml, Iopromide, Bayer Healthcare) was administered at a flow rate of 5 ml/s, followed by a 40 ml saline solution.

**Table 1:** Scan parameters

Scan parameters	
CT plan scan (calcium score scan)	
Tube voltage (KV)	100
Tube current (mAs)	414 (350, 562)
Slice thickness (mm)	0.625
Slice gap (mm)	0.625
FOV (cm)	21.2
Estimated effective dose (mSv)	0.67 (0.54, 0.88)
CCTA scan	
Tube voltage (KV)	100
Tube current (mAs)	599 (599, 599)
Slice thickness (mm)	0.625
Slice gap (mm)	0.625
FOV (cm)	21.2
Retrospective gating	Yes
Scan trigger mode	Bolus tracking
Estimated effective dose (mSv)	2.87 (2.68, 3.05)

*FOV* Field of view

### CACS and Diameter Stenosis (DS) assessment

To quantify coronary plaque calcification, the Agatston score was calculated for each vessel by using post-processing workstation (Advantage Workstation, version 4.7, GE Healthcare, Milwaukee, USA). DS was divided into: 0% no stenosis, 1–24% minimal stenosis, 25–49% mild stenosis, 50–69% moderate stenosis, 70–99% severe stenosis and 100% occlusion on vessel-based analysis [21]. DS ≧ 50% was considered as obstructive CAD.

### FFR_CT_ analysis

**Fig. 1:**
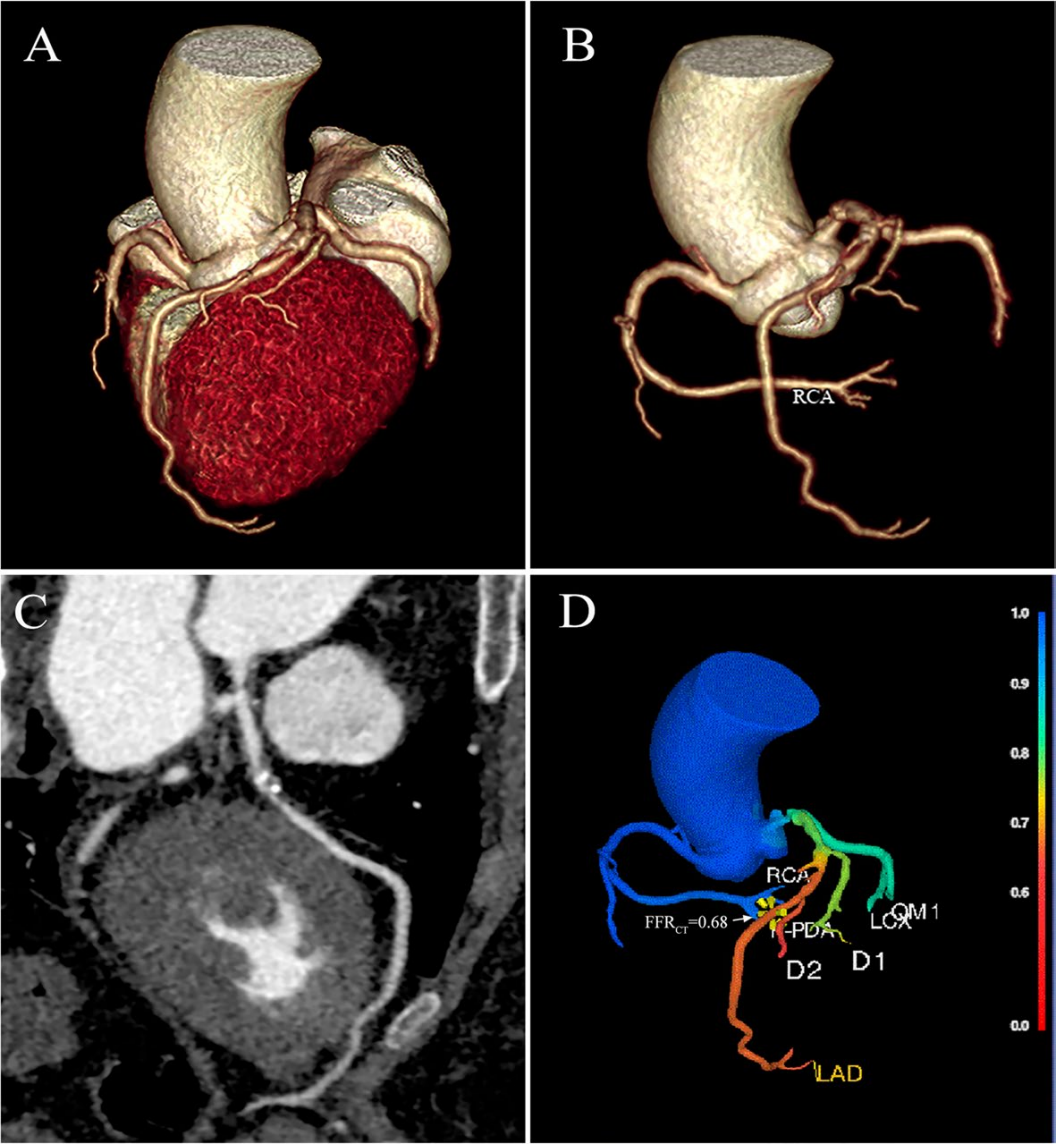
Example case. The left anterior descending artery contained mixed plaques with functional ischemia: 3-dimensional (volume rendering) (**A, B**), curved planar reconstruction (**C**) and coronary computed tomography angiography derived fractional flow reserve [the measurement location (yellow marker and white arrow): 2 cm distal to the stenosis in plaque artery]. (**D**)

All FFR_CT_ values were calculated with an automated software (“Shukun-FFR” software from Shukun [Beijing] Technology Co., Ltd). As described in the study [22], the coronary arteries segmentation model and the computational fluid dynamics (CFD) simulation model were used in the "Shukun-FFR" software. The calculation process was as follows: firstly, coronary artery from CCTA image was segmented with a modified V-Net to generate a coronary tree; then, FFR_CT_ values of all points in coronary arteries were calculated automatically by the final reduced-order CFD model computing the flow and pressure of blood. FFR_CT_ was measured at 2 cm distal to the stenosis in plaque artery, while the measuring position was located at the end of the vessel in plaque-free artery (at least ≥ 1.5 mm in diameter). In the case of multiple stenoses in a single vessel, the distal end of the farthest lesion was measured. FFR_CT_ ≤ 0.80 was defined as haemodynamically significant coronary stenosis (Fig. 1).

### PCAT segmentation

PCAT segmentation of the non-contrast CT images was performed using Perivascular Fat Analysis Tool software (v1.1.0.) As previously reported, we traced the proximal 40 mm segments of the left anterior descending artery (LAD), left circumflex artery (LCX) and the proximal 10–50 mm segments of the right coronary artery (RCA) [14]. Region of interest (ROI) was delineated manually by one experienced radiologist and supervised by another experienced radiologist. PCAT was defined as the adipose tissue within a radial distance from the outer vessel wall equal to the diameter of the vessel, with the attenuation between -190 to -30 HU [14]. PCAT could be segmented automatically by the software.

### Feature extraction and selection

Radiomics features were extracted using the “Calculate Radiomics” module in Perivascular Fat Analysis Tool software. 1691 radiomic features were extracted as follows: (1) first-order features; (2) size and shape features; (3) texture features. Texture features included Gray Level Co-occurrence Matrix (GLCM), Gray Level Dependence Matrix (GLDM), Gray Level Size Zone Matrix (GLSZM), Gray Level Run Length Matrix (GLRLM), Neighboring Gray Tone Difference Matrix (NGTDM). Wavelet transform images were generated by 8 different combinations of high and low frequency bands in 3 directions (x, y, z), providing high-dimensional multi-frequency information. Sigma values of Laplacian of Gaussian (LoG) filtered images were set to 1, 2, 3, 4 and 5 mm respectively. Nonlinear strength transformation of image voxel included square, square root, logarithm and exponential operations.

Minimum redundancy maximum relevance (mRMR) and least absolute shrinkage and selection operator (LASSO) were applied to reduce the dimensionality of high-dimensional data and screen the radiomics features. To obtain an optimal feature subset, a 5-fold cross-validation was used to choose the optimal λ and features with non-zero coefficient were finally selected.

### Fat Attenuation Index (FAI) achievement

**Fig. 2:**
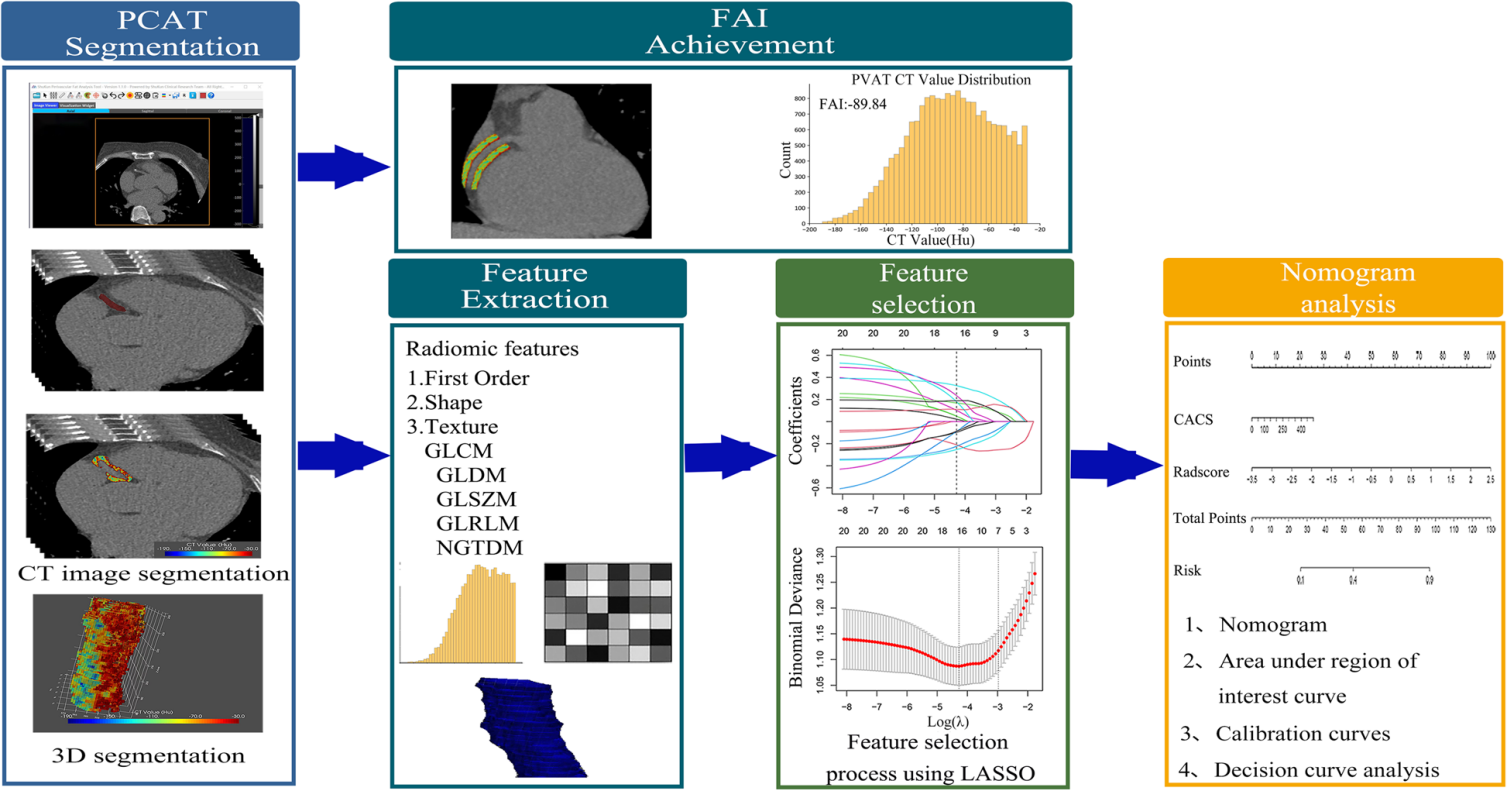
The workflow for FAI achievement and radiomics nomogram development

After PCAT segmentation was finished on non-contrast CT images, FAI could be achieved simultaneously with radiomic feature extraction using Perivascular Fat Analysis Tool software (Fig. 2). FAI was defined as the mean CT attenuation of PCAT [14].

### Model construction and validation

A radiomics signature was built using the LASSO logistic regression model and a linear formula was used to calculate the score value of the radiomics signature (Radscore). Based on the training cohort, univariate and multivariable logistic regression was conducted to select the independent predictors among CACS, FAI and Radscore. These independent predictors were used to establish radiomics nomogram to predict haemodynamically significant coronary stenosis.

The sensitivity, specificity, accuracy, positive predictive value (PPV), negative predictive value (NPV), receiver operating characteristic (ROC) curves were used to evaluate the predictive ability of the models. Areas under ROC curves (AUCs) among models were compared by DeLong test. Hosmer–Lemeshow test and calibration curve were used to analyze whether the difference between the predicted risk rate and observed probability was statistically significant. The net benefits of the model and its clinical utility were evaluated by decision curve analysis (DCA). The workflow of nomogram was shown in Fig. 2.

### Statistical analysis

Statistical analyses were performed using SPSS Statistics (version 26.0) and R software (R version 4.05 and R Studio version 4.0). *P* value < 0.05 was regarded to be statistically significant. Means, standard deviations, or the median and interquartile range (IQR) were used to express continuous variables depending on whether the data was normal distribution. Categorical variables were expressed as numbers (%). Independent sample t-test or Wilcoxon rank sum test was applied to compare the quantitative dates. Chi-square test was used for qualitative variables. “glmnet” package was performed to implement the LASSO regression. “RMS” package was used to build multivariate logistic regression, nomogram, and calibration curves. “pROC” package was used to analyze ROC curves.

## Results

### Clinical characteristics

Totally, 514 vessels (training cohort: 360; validation cohort: 154) in 215 patients were included in this study. 157 vessels stenoses (30.5%) were significant based on FFR_CT_. There were significant differences between the FFR_CT_ > 0.8 group and FFR_CT_ ≤ 0.8 group in terms of CACS and lesion distributions. Patient and lesion characteristics were shown in Tables 2 and 3.

**Table 2:** Patient characteristics

Characteristics	Overall (*n* = 215)
Age (years), median (IQR)	62 (55–68)
BMI (kg/m^2^) median (IQR)	24.2 (22.3–26.0)
Female, n (%)	123 (57.2%)
Risk factors, n (%)	
Hypertension, n (%)	157 (73.0%)
Hyperlipidemia, n (%)	81 (37.7%)
Smoking, n (%)	50 (23.3%)
Drinking, n (%)	41 (19.1%)
Family history of CAD, n (%)	9 (4.2%)

*IQR* Interquartile range, *BMI* Body mass index, *CAD* Coronary artery disease

**Table 3:** Lesion characteristics in no-contrast CT

Characteristics	FFR > 0.8 (*n* = 357)	FFR ≤ 0.8 (*n* = 157)	*P* value
FFR_CT,_ median (IQR)	0.92 (0.88–0.96)	0.72 (0.66–0.77)	< 0.001
CACS, Agatston units, (IQR)	0(0–25.43)	89.48(1.93–197.63)	< 0.001
Lesion distributions, n (%)			< 0.001
LAD	112 (31.4)	93 (59.2)	
RCA	153 (42.9)	54 (34.4)	
LCX	92 (25.7)	10 (6.4)	
FAI (Hu), mean ± SD	-85.77 ± 6.45	-84.59 ± 6.88	0.062

*FFR* Fractional flow reserve, *IQR* Interquartile range, *CACS* Coronary artery calcium score, *LAD* Left anterior descending artery, *RCA* Right coronary artery, *LCX* Left circumflex artery, *FAI* Fat attenuation index, *HU* Hounsfield units, *SD* Standard deviation

### Radiomics model construction and evaluation

**Fig. 3:**
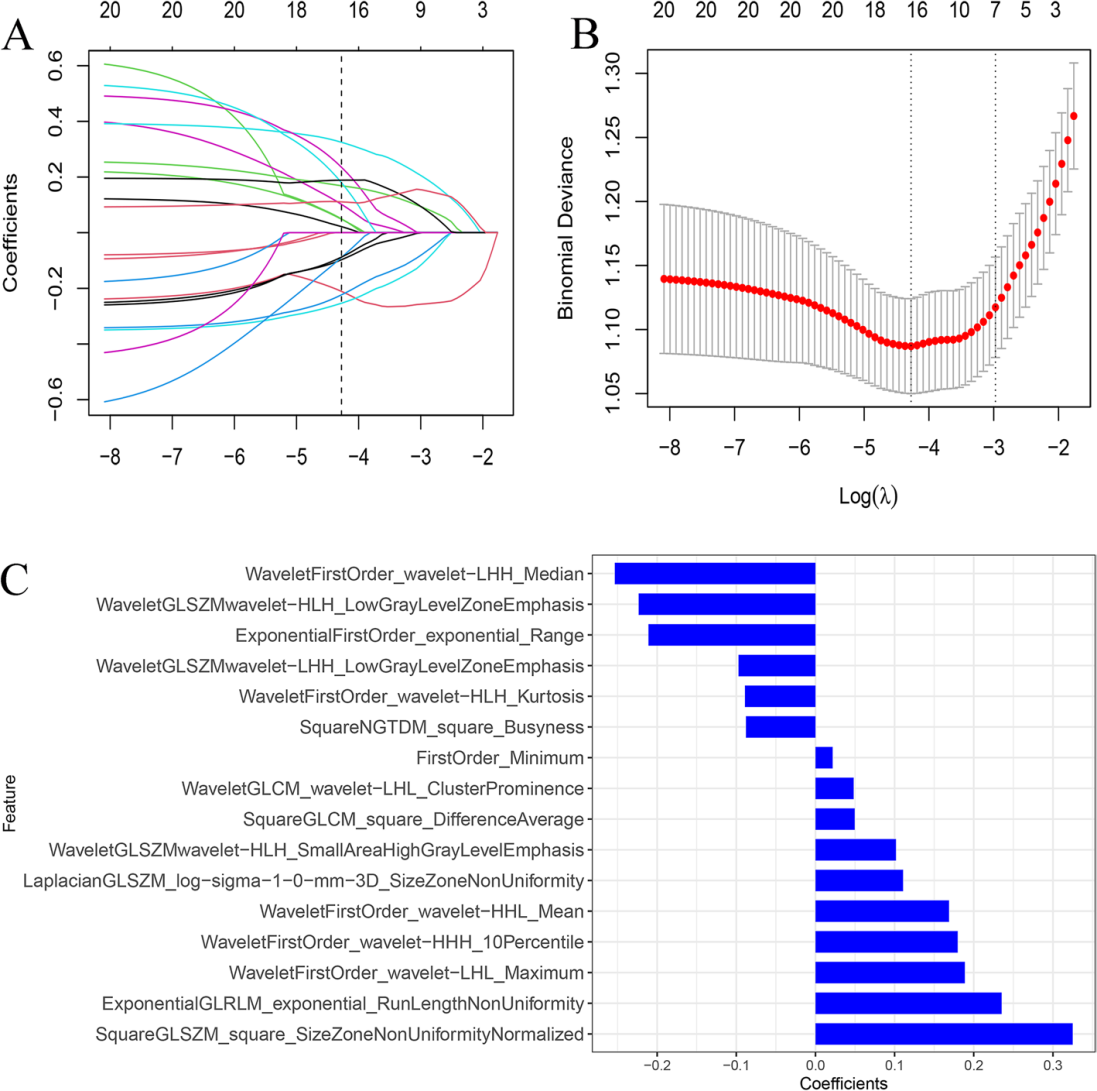
Feature selection process. The y axis represented LASSO coefficient profiles of the radiomics features and the lower x-axis indicated the log lambda (λ) (**A**). Sixteen radiomic features were selected to calculate radiomics score (**B**). The regression coefficients in the selected radiomics features (**C**)

**Fig. 4:**
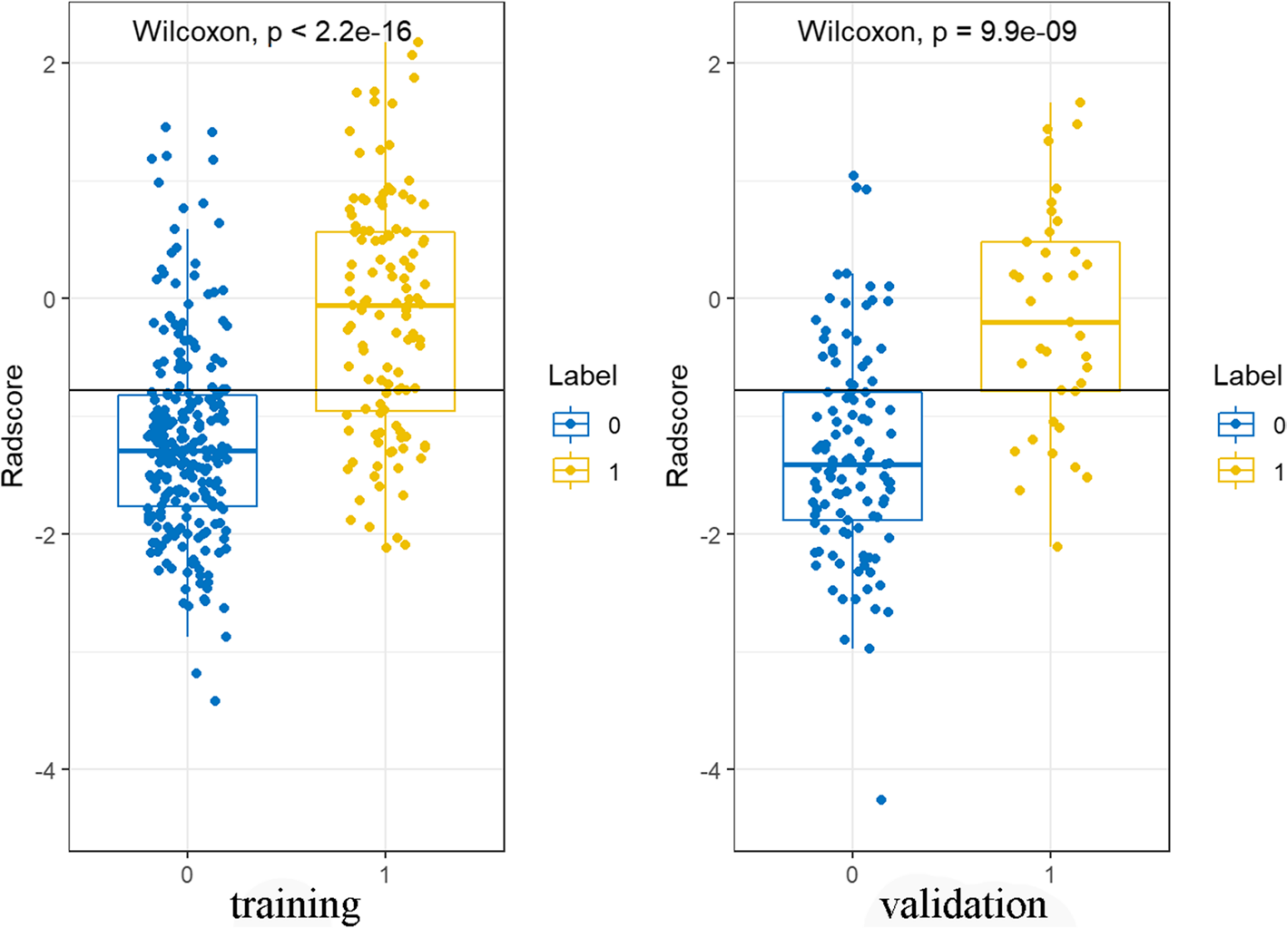
Distribution of the Radscore in the training and validation cohorts indicated that Radscore had an excellent ability to predict haemodynamically significant coronary stenosis in patients with type 2 diabetes. Coronary arteries without functional ischemia (blue); Coronary arteries with functional ischemia (yellow)

After the features extracting, mRMR was performed to remove redundant features, and 20 features were retained. Based on the training cohort, a total of sixteen predictive features were selected by LASSO regression to build the radiomics signature (Fig. 3A-C). The formula was as follows:

Radscore = 0.022 * FirstOrder_Minimum + -0.211 * ExponentialFirstOrder_exponential_Range + 0.05 * SquareGLCM_square_DifferenceAverage + -0.223 * WaveletGLSZMwavelet-HLH_LowGrayLevelZoneEmphasis + -0.253 * WaveletFirstOrder_wavelet-LHH_Median + 0.102 * WaveletGLSZMwavelet-HLH_SmallAreaHighGrayLevelEmphasis + -0.097 * WaveletGLSZMwavelet-LHH_LowGrayLevelZoneEmphasis + 0.048 * WaveletGLCM_wavelet-LHL_ClusterProminence + -0.089 * WaveletFirstOrder_wavelet-HLH_Kurtosis + 0.325 * SquareGLSZM_square_SizeZoneNonUniformityNormalized + 0.235 * ExponentialGLRLM_exponential_RunLengthNonUniformity + -0.088 * SquareNGTDM_square_Busyness + 0.169*WaveletFirstOrder_wavelet-HHL_Mean + 0.18 * WaveletFirstOrder_wavelet-HHH_10Percentile + 0.189 * WaveletFirstOrder_wavelet-LHL_Maximum + 0.111 * LaplacianGLSZM_log-sigma-1–0-mm-3D_SizeZoneNonUniformity + -0.859. The Radscore distribution in both cohorts were shown in Fig. 4. The optimum cutoff value of Radscore in training cohort was -0.78.

**Fig. 5:**
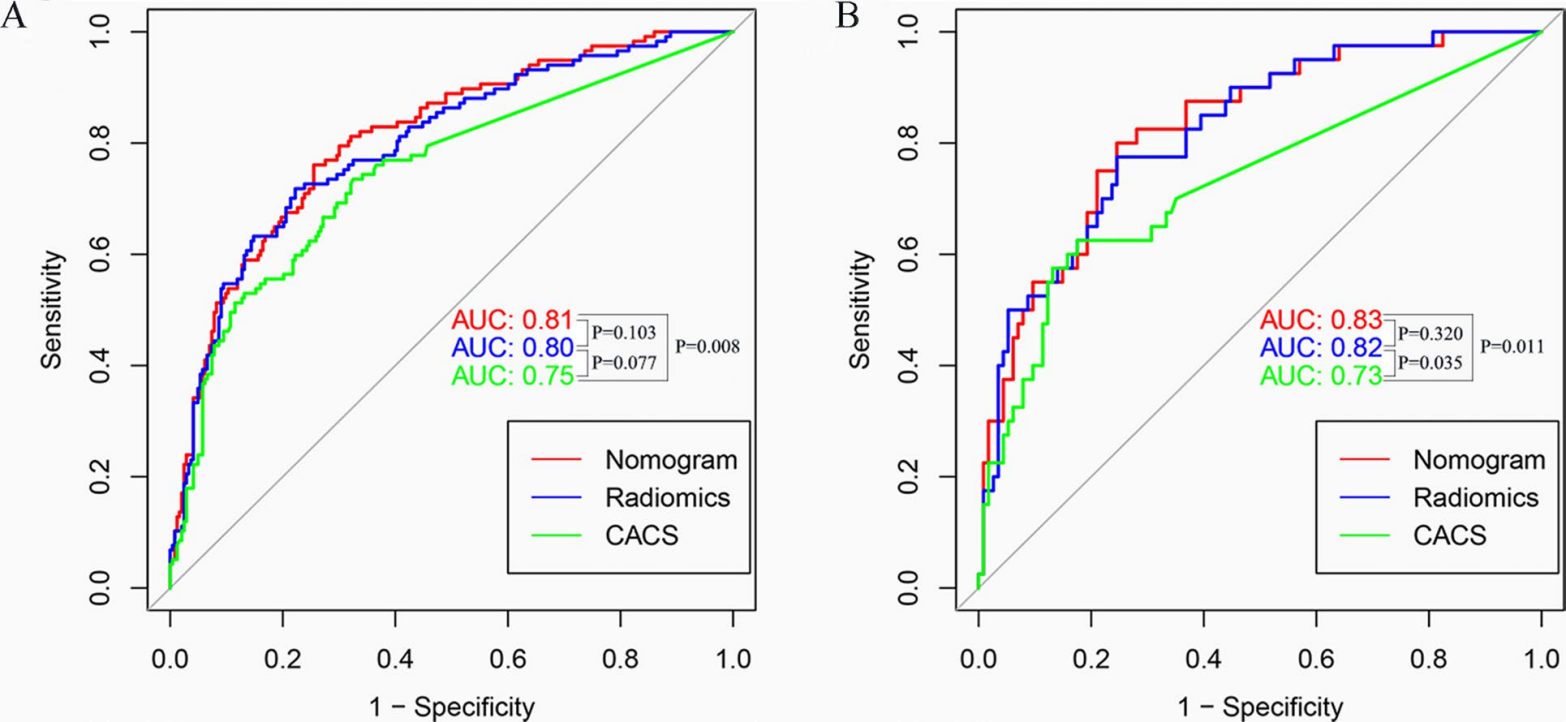
AUCs of CACS, Radscore and radiomics nomogram for predicting haemodynamically significant coronary stenosis in patients with type 2 diabetes in each cohort. AUC, area under receiver operating characteristic curve

Radscore showed good performance (training: AUC = 0.80; 95% CI: 0.75–0.85; validation: AUC = 0.82; 95% CI: 0.75–0.90) to predict haemodynamically significant coronary stenosis **(**Fig. 5A-B**)**. Radscore yielded significantly higher AUC than FAI in training (AUC 0.80 vs 0.54, *P* < 0.001) and validation cohorts (AUC 0.82 vs 0.57, *P* < 0.001).

### Radiomics nomogram construction

**Fig. 6:**
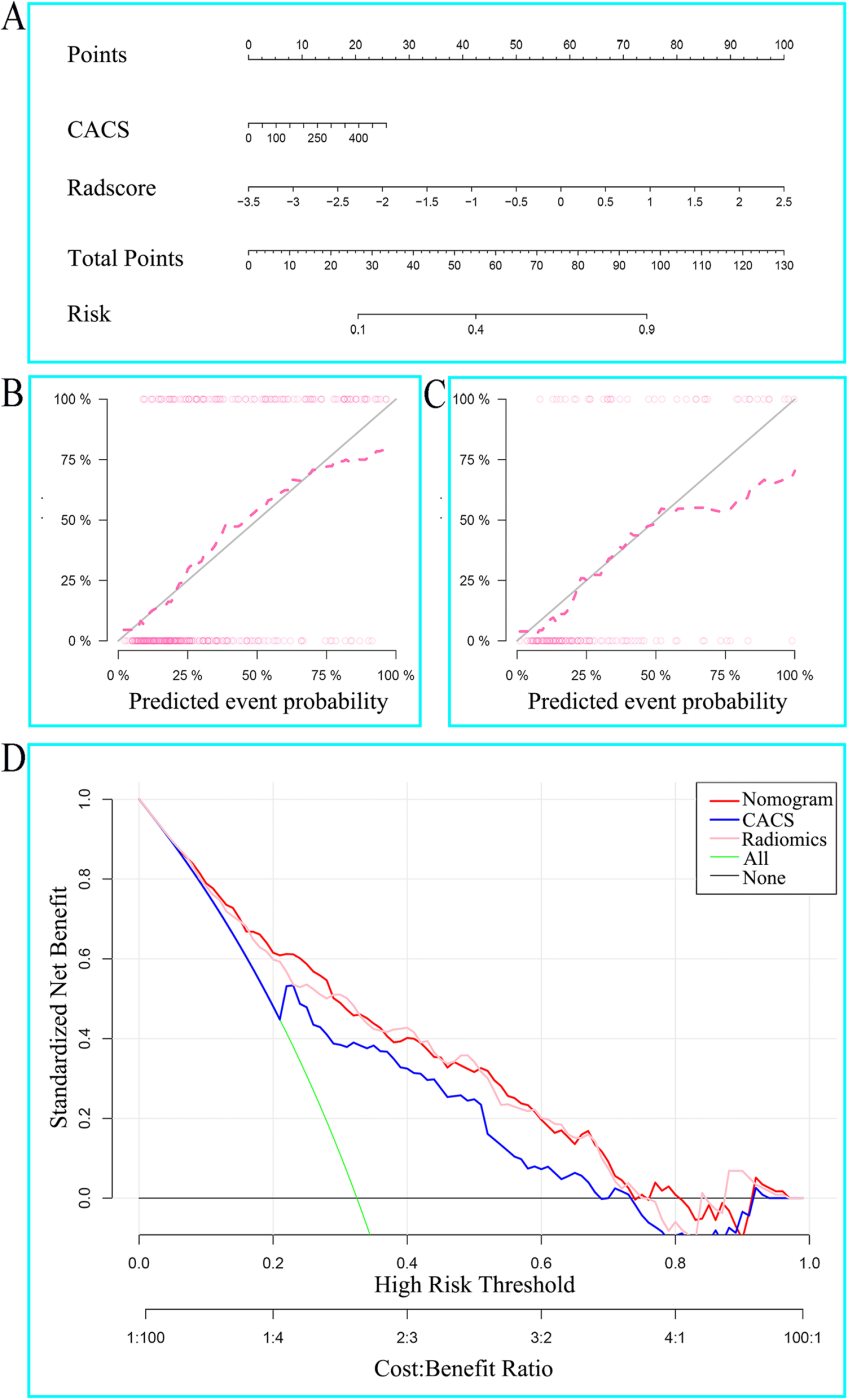
The nomogram was constructed with the Radscore and CACS (**A**). Calibration curves of the radiomics nomogram in the training (**B**) and validation (**C**) cohorts. Decision curves indicated that radiomics nomogram and Radscore basically had higher clinical application value than CACS when risk threshold between 0.1 and 0.8 (**D**)

According to univariate and multivariable regression, CACS and Radscore were identified as the independent predictors for haemodynamically significant coronary stenosis in the training cohort (Table 4). Then, Radiomics nomogram was constructed correspondingly (Fig. 6A). The radiomics nomogram showed excellent performance on prediction for haemodynamically significant coronary stenosis (AUC, 0.81; 95%CI, 0.76–0.86), which was confirmed in the validation cohort (AUC, 0.83; 95%CI, 0.76–0.90) (Fig. 5A-B**)**. Hosmer–Lemeshow test (*P* > 0.1) and the calibration curve showed good calibration performance between the predicted and observed results in the training and validation cohorts (Fig. 6B-C). Clinical usefulness was evaluated by DCA (Fig. 6D). Radiomics nomogram and Radscore basically had higher clinical application value than CACS when risk threshold was between 0.1 and 0.8.

**Table 4:** Univariate and multivariate logistic regression identified significantly independent factors in the training cohort to construct radiomics nomogram

Indicators	Univariate analysis				Multivariable analysis			
	OR	95% CI		*P* value	OR	95% CI		*P* value
		Lower	Upper			Lower	Upper	
CACS	1.01	1.01	1.01	< 0.001	1.00	1.00	1.01	0.023
FAI	1.02	0.99	1.06	0.241	-	-	-	-
Radscore	3.46	2.58	4.65	< 0.001	2.84	2.04	3.96	< 0.001

*OR* Odds ratio, *CI* Confidence interval, *FAI* Fat attenuation index, *CACS* Coronary artery calcium score

### FAI in different DS categories

The FAI values in different DS categories were shown in Table 5. There was no statistical difference in FAI among the six categories (*P* = 0.524). The FAI was no statistically different between obstructive CAD and non-obstructive CAD (FAI -85.39 ± 6.83 HU vs -85.42 ± 6.50 HU, *P* = 0.962).

**Table 5:** The correlation between FAI and CAD-RADS

Diameter stenosis	FAI (HU)	*P* value
0%	-85.24 ± 6.84	0.524
1–24%	-85.38 ± 5.81	
25–49%	-85.71 ± 6.37	
50–69%	-86.27 ± 6.17	
70–99%	-84.68 ± 7.00	
100%	-82.82 ± 11.22	

*FAI* Fat attenuation index, *HU* Hounsfield units

### Comparison of CACS, Radscore and radiomics nomogram

The predictive performances for haemodynamically significant coronary stenosis among CACS, Radscore and radiomics nomogram were shown in Table 6 and Fig. 5.

**Table 6:** The performance of CACS, Radscore and radiomics nomogram

Models	Cohorts	AUC (95% CI)	Accuracy	Sensitivity	Specificity	PPV	NPV
CACS	Training cohort	0.75(0.69–0.80)	69.4%	73.5%	67.5%	52.1%	84.1%
	Validation cohort	0.73(0.64–0.82)	71.4%	62.5%	74.6%	46.3%	85.0%
Radscore	Training cohort	0.80(0.75–0.85)	75.8%	71.8%	77.8%	60.9%	85.1%
	Validation cohort	0.82(0.75–0.90)	75.3%	75.0%	75.4%	51.7%	89.6%
Radiomics nomogram	Training cohort	0.81(0.76–0.86)	75.0%	76.1%	74.5%	58.9%	86.6%
	Validation cohort	0.83(0.76–0.90)	76.0%	75.0%	76.3%	52.6%	89.7%

*CACS* Coronary artery calcium score, *AUC* Area under receiver operating characteristic curve, *PPV* Positive predictive value, *NPV* Negative predictive value

In the validation cohort, Radscore achieved significantly higher AUC than CACS (AUC 0.82 vs 0.73, *P* = 0.035). Radiomics nomogram also achieved significantly higher AUC than CACS (AUC 0.83 vs 0.73, *P* = 0.011), while there was no statistical difference between radiomics nomogram and Radscore (AUC 0.83 vs 0.82, *P* = 0.320).

## Discussion

In this study, a non-contrast CT-based radiomics nomogram of PCAT by integrating radiomics features and CACS was established and validated. Our results demonstrated the radiomics nomogram had excellent predictive performance for haemodynamically significant coronary stenosis and could be a promising noninvasive method to optimize risk stratification and guide treatment strategies in T2DM patients.

It is of especially crucial to find new and efficient methods to predict high-risk CAD with myocardial ischemia both in time and extent, on account of that CAD in T2DM patients is often asymptomatic and undiagnosed until acute myocardial infarction occurs [23] and early detection of high-risk CAD can provide an opportunity for early intervention and prevent MACE. FFRCT is widely used for prediction of function myocardial ischemia, influence on treatment decision making and prognostic evaluation of clinical outcome, however the technology is not suitable for screening of high-risk CAD especially for asymptomatic because of relative low incidence, low MACE rate, high cost, adverse side effects and high radiation exposure [24, 25]. Our study provides a novel noninvasive, practical and economical tool for predicting haemodynamically significant coronary stenosis in patients with T2DM, with low radiation dose and iodine avoiding, especially suitable for silent CAD. In our study, the radiomics nomogram achieved accurate prediction with AUC of 0.83 in the validation cohort. The median estimated effective dose of no-contrast CT in the present study is 0.67 mSv, which is consistent with our goal to screen high-risk CAD with a relative lower radiation dose, iodine avoiding and costs reduction.

Plaque development and lumen stenosis are caused by vascular inflammation, inducing endothelial dysfunction and impaired vasodilation, which may reduce the distal flow reserve and result in functional ischemia [26, 27]. Previous research demonstrated that FAI could identify coronary inflammation by capturing changes in perivascular fat attenuation, and also had value in predicting hemodynamic significance of coronary stenosis with AUC of 0.83 [28, 29]. However, in this study, the predictive performance of FAI was insufficient to identify haemodynamically significant coronary stenosis. It was controversial whether FAI can identify haemodynamically significant coronary stenosis, and the present study was consistent with previous studies with AUC from 0.55–0.67 [18, 28–30]. There might be related with following reasons: first, different CT acquisition parameters had influence on FAI values; second, we traced the proximal 40 mm segments of LAD and LCX and the proximal 10–50 mm segment of RCA, while other studies traced the specific stenosis in CCTA [29]; third, the FAI analysis in above studies was based on CCTA, while we measured FAI on non-contract CT; forth, FAI might be changed by treatments [16].

Haemodynamically significant coronary stenosis caused by vascular inflammation [26, 27], might lead to alterations in the microenvironment and tissue components within the PCAT. Radiomics can extract thousands of quantitative imaging features from medical image data, and construct prediction models by selecting the most valuable features [17]. In our study, the Radscore was an independent predictor for functional ischemia. It was derived from the sixteen most contributive radiomic features extracted from PACT, including seven first-order features and nine texture features, without shaped features. Only one feature was extracted from the original images, nine features from wavelet, one feature from LoG filtered images and five from nonlinear strength transformation. First-order features reflect the intensity features containing gray histogram information of PCAT, and the texture features reflect the heterogeneity of PCAT. From the radiomics model, we found that, the PCAT of the positive FFR_CT_ case showed high heterogeneity (ExponentialGLRLM_exponential_RunLengthNonUniformity, SquareGLSZM_square_SizeZoneNonUniformityNormalized and LaplacianGLSZM_log − sigma − 1 − 0 − mm − 3D_SizeZoneNonUniformity), and a small proportion of lower gray-level values (WaveletGLSZMwavelet − HLH_LowGrayLevelZoneEmphasis and WaveletGLSZMwavelet − LHH_LowGrayLevelZoneEmphasis). These features mainly reflected the confusion, complexity and variability of PCAT and were potentially captured by PCAT radiomics features instead of PCAT attenuation. Radiomics signature is not only reflect the acute inflammation, but also describes fibrosis and vascularity in adipose tissue induced by chronic coronary inflammation [16]. Moreover, fat radiomics signature reveals persistently structural changes in PVAT and a corresponding residual risk not confounded by medications taking or other acute processes. Hence, Radscore performed better than FAI to identify haemodynamically significant coronary stenosis in patients with T2DM.

Hyperglycemia, insulin resistance and excess fatty acids in T2DM enhance oxidative stress, destroy protein kinase C signaling and increase advanced glycation end products which result in vascular inflammation, vasoconstriction and atherogenesis [31, 32]. Complex mechanism leads to release of osteoprogenitor cells form the bone marrow into the circulation, promoting coronary intimal calcification [33, 34]. Consistent with above studies [28], coronary artery calcification in the present study was shown to be an independent predictor for haemodynamically significant coronary stenosis in T2DM. Radiomics analysis has higher predictive ability than CACS and is particularly important to screen high-risk patients without severe coronary calcification in early T2DM patients.

However, there are several limitations in this study. First, image acquisition was acquired from the same CT manufacturer in order to ensure the image uniformity, which needed to verify the generalization of the present findings in other manufacturers further. Second, a multi-center study with a larger sample count should be performed for further research, since this single-center retrospective study lacked external validation. Third, the sample size was insufficient, not allowing for subgroup study, especially for patients in “gray-zone lesions”. Fourth, part of LCXs were not analyzed because of anatomic variation and difficult delineation. Fifth, the models demonstrated higher predictive value in the validation cohort than in the training cohort. The reason for this result may be that the data set was not large enough, even though the training and test sets were randomly assigned, the distribution of the training and test sets were somewhat uneven. Another possible reason was that there might be underfitting in the training cohort and led to the lowering predictive value of the training cohort.

## Conclusion

In conclusion, a radiomics nomogram of PCAT based on non-contrast CT has excellent performance for discriminating coronary functional ischemia, which may potentially  become a noninvasive and economical method for predicting and screening high-risk CAD in patients with T2DM.

## Data Availability

The datasets analysed during the current study are available from the corresponding author on reasonable request.

## Abbreviations

CT: Computed tomography
PCAT: Pericoronary adipose tissue
FFR: Fractional flow reserve
T2DM: Type 2 diabetes mellitus
CCTA: Coronary computed tomography angiography
FAI: Fat attenuation index
LASSO: The least absolute shrinkage and selection operator
mRMR: The minimum redundancy maximum relevance
CACS: Coronary artery calcium score
AUC: Area under receiver operating characteristic curve
DCA: Decision curve analysis
CAD: Coronary artery disease
MACE: Major adverse cardiac events
DS: Diameter stenosis
CCS: Calcium score scan
PCI: Percutaneous coronary intervention
CABG: Coronary artery bypass grafting
AS: Agatston score
CFD: Computational fluid dynamics
RCA: Right coronary artery
LAD: Left anterior descending artery
LCX: Left circumflex artery
ROI: Region of interest
GLDM: Gray Level Dependence Matrix
GLCM: Gray Level Co-occurrence Matrix
GLRLM: Gray Level Run Length Matrix
GLSZM: Gray Level Size Zone Matrix
NGTDM: Neighboring Gray Tone Difference Matrix
LoG: Laplacian of Gaussian
ROC: Receiver operating characteristic
IQR: Interquartile range
FOV: Field of view
HU: Hounsfield units
BMI: Body mass index
SD: Standard Deviation
PPV: Positive predictive value
NPV: Negative predictive value
